# Effect of a high-fat diet and iron overload on erythropoiesis in mice

**DOI:** 10.1016/j.bbrep.2025.101919

**Published:** 2025-02-01

**Authors:** Joe Varghese, Jithu James Varghese, Molly Jacob

**Affiliations:** Department of Biochemistry, Christian Medical College, Vellore, Tamil Nadu, India^1^

**Keywords:** Terminal erythroid differentiation, Hyperinsulinemia, Hepcidin, High-fat diet, Iron overload

## Abstract

**Background:**

Insulin and iron availability stimulate and regulate erythropoiesis, respectively. The effects of hyperinsulinemia and/or iron overload on erythroid differentiation are unclear.

**Methodology:**

Male C57Bl/6J wild-type (WT) mice were fed a high-fat diet (HFD) (to produce hyperinsulinemia) or a control diet (CD) for varying periods (4–24 weeks). Hepcidin knock-out (*Hamp1*^*−/−*^) mice (which are iron-overloaded) were fed CD or HFD for 24 weeks. Terminal erythroid differentiation (TED) in the bone marrow (BM) from these mice was analyzed by flow cytometry. Hematological parameters were estimated in peripheral blood.

**Results:**

HFD-feeding of WT mice did not significantly affect erythroid precursors in the BM or hematological parameters. However, these mice had a significantly higher reticulocyte population in the BM than those fed CD (at all time points studied). Values of hematological parameters were higher in *Hamp1*^*−/−*^ mice than WT mice, at 24 weeks of feeding (irrespective of diet type), indicating increased erythropoiesis. Early erythroid precursors in the BM were higher in HFD-fed *Hamp1*^*−/−*^ mice than those fed CD.

**Conclusions:**

HFD-feeding in WT mice resulted in increases in the proportion of reticulocytes in the bone marrow; maturation of the early erythroid precursors was not significantly affected. In *Hamp1*^*−/−*^ mice, HFD-feeding increased the number of early erythroid precursors.

## Highlights

• The impact of hyperinsulinemia and iron overload on erythropoiesis is unclear.
• Wild-type and hepcidin knockout mice were fed high-fat diet (HFD) or control diet.
• Terminal erythroid maturation (TED) was assessed by flow cytometry.
• HFD-feeding of wild-type mice resulted in increased reticulocytes in the bone marrow.
• In Hamp1−/− mice, HFD-feeding increased the number of early erythroid precursors.

## 1 Introduction

Erythropoiesis refers to the differentiation of hematopoietic stem cells (HSCs) to form reticulocytes before their release into the circulation. In mice, erythropoiesis consists of two phases: the formation of early erythroid progenitors and terminal erythroid differentiation (TED). In the first phase, HSCs proliferate and differentiate to form early erythroid progenitor cells, called burst-forming units (BFU-E) and colony-forming units (CFU-E) [1]. The earliest morphologically recognizable erythroid precursor, the proerythroblast (pro-E), is derived from the CFU-E. In the second phase (terminal erythroid differentiation or TED), pro-E undergoes three cycles of mitosis, resulting in the sequential formation of basophilic (BE), polychromatic (PE), and orthochromatic (OE) erythroblasts. Accordingly, each pro-E gives rise to 2 BEs, which, in turn, forms 4 PEs and then 8 OEs. Therefore, TED is typically characterized by the presence of pro-E, BE, PE and OE in the ratio of 1:2:4:8 in the bone marrow [2,3].

Hyperinsulinemia, secondary to insulin resistance (IR), is a characteristic feature of type 2 diabetes mellitus (T2DM) and the metabolic syndrome [4]. Multiple studies have shown an association between IR and elevated levels of hematological parameters, such as hemoglobin, RBC counts, and hematocrit, suggesting enhanced erythropoiesis in this setting [[5], [6], [7], [8], [9], [10], [11]]. *In vitro* clonogenic assays, using isolated hematopoietic progenitor cells, have shown that insulin stimulates the proliferation and differentiation of early erythroid progenitors, BFU-E and CFU-E [[12], [13], [14], [15]]. Therefore, it has been postulated that the enhanced erythropoiesis associated with IR may be mediated by hyperinsulinemia [5]. However, this hypothesis has not been tested by *in vivo* studies.

The availability of iron is a critical regulator of erythropoiesis [16]. Iron deficiency decreases erythropoietin production in the kidney, and also directly impairs erythroid maturation in the bone marrow [17]. However, the effect of high levels of body iron on erythropoiesis has not been investigated. Hepcidin, a peptide hormone predominantly synthesized and secreted by the liver, regulates systemic iron homeostasis [18]. Mice that lack the hepcidin gene (hepcidin knock-out or *Hamp1*^*−/−*^ mice), develop iron overload as a result of uninhibited iron absorption from the intestine [19]. *Hamp1*^*−/−*^ mice are, therefore, commonly used as models of iron overload [20].

We have shown earlier that IR induced by high-fat diet (HFD)-feeding in wild-type mice resulted in hyperinsulinemia and dysregulation of systemic iron homeostasis. These mice were found to have decreased liver iron stores and increased iron content in the adipose tissue [21]. *Hamp1*^*−/−*^ mice, on the other hand, developed less insulin resistance and did not develop hyperinsulinemia in response to HFD-feeding [22]. It is not known whether these changes seen in insulin sensitivity and iron homeostasis in these mice affect erythropoiesis. Given the above background, we attempted to determine the effects of HFD-feeding on erythropoiesis in wild-type mice and iron-overloaded *Hamp1*^−/−^ mice.

## 2 Methods

### 2.1 Animals

All animal experiments were carried out with the approval of the Institutional Animal Ethics Committee at Christian Medical College, Vellore, India (IAEC No. 14/2013 and 8/2014), in accordance with the regulations of the Committee for Control and Supervision of Experiments on Animals (CCSEA), Government of India, and the guidelines given in Guide for the Care and Use of Laboratory Animals, 8th edition [23].

Male C57Bl/6J mice (aged 8 weeks) were fed either a control diet (CD) (Research Diets, Inc., USA, #D12450J, with 10 % of total calories derived from fat) or a high-fat diet (HFD) (Research Diets, Inc., USA, #D12492, with 60 % of total calories derived from fat) for 4, 8, 12, 16, 20 or 24 weeks, as described earlier [21]. Male hepcidin knock-out (*Hamp1*^*−/−*^) mice (on a C57Bl/6J background) were also fed the CD or HFD for 24 weeks, as described earlier [22]. We used terminal inhalational anaesthesia with isoflurane as the method of euthanasia.

### 2.2 Flow cytometric analyses of erythroid precursors in the bone marrow

After euthanizing the mice at the end of the feeding periods, the tibias and femurs from both sides were removed and the bone marrow from each was flushed out and single-cell suspensions were prepared [24]. Flow cytometric analysis of terminal erythroid differentiation was carried out using FACS Aria III flow cytometer (BD Biosciences, USA), as described earlier [2,3,24]. Briefly, single-cell suspensions obtained from the bone marrow were stained with *anti*-TER119, anti-CD71 and anti-CD44 antibodies. TER119 is a marker of erythroid lineage. TER119-positive cells were gated, based on CD71 (TfR1) [2,24] or CD44 [3] expression, to resolve them into various stages of erythroid maturation. Details of the protocols used for staining, antibodies used, controls used, etc. are described in detail under Supplementary Methods. Data obtained were analyzed using FlowJo, version 10.4 (FlowJo, LLC, USA).

We used two strategies to study the maturation of erythroid precursors. The first was based on CD71 expression and cell size (forward scatter [FSC]) [[24], [25], [26]]. CD71 (or transferrin receptor 1 [TfR1], which is involved in cellular uptake of iron from transferrin in circulation) expression is highest in pro-erythroblasts and basophilic erythroblasts; it declines to lower levels in reticulocytes and is not detectable in mature erythrocytes [27]. Ter119-positive cells were resolved into four distinct stages (called ProE, EryA, EryB and EryC) based on changes in surface expression of CD71 [26] and in cell size [25], as shown in Suppl Fig. 1. Pro-erythroblasts (ProE) are CD71^high^-Ter119^intermediate^ cells, representing the earliest erythroid precursors. EryA (CD71^high^-Ter119^high^-FSC^high^) represents the basophilic and polychromatic erythroblasts (early erythroid precursors). EryB (CD71^high^-Ter119^high^-FSC^low^) represents the orthochromatic erythroblasts and reticulocytes (late erythroid precursors) and EryC (CD71^low^-Ter119^high^-FSC^low^) represents mature RBCs [2].

In the second strategy, TER119-positive cells were resolved, based on expression of CD44 and FSC [3]. CD44, a ubiquitously expressed type-1 transmembrane glycoprotein [28], is highly expressed in early erythroblasts; its expression progressively declines as the erythroblasts mature. Using this method, six distinct populations were defined, as shown in Suppl Fig. 2 [2,25]. Here, sub-population (Pop) I (CD44^high^–FSC^high^ cells) represents the pro-erythroblasts. Pop II, III, IV and V were categorized based on decreasing CD44 expression and FSC. Pop-II and Pop-III (polychromatic and basophilic erythroblasts, respectively) represent early erythroid precursors, while IV and V (orthochromatic erythroblast and reticulocytes, respectively) represent the late erythroid precursors. This strategy has the advantage of resolving individual erythroblast populations with greater precision than is achieved by CD71/FSC [2]. Single-stained controls and fluorescence-minus-one (FMO) controls are shown in Suppl Figs. 3 and 4, respectively.

### 2.3 Hematological parameters

Hematological parameters in mouse peripheral blood samples were measured using the ABX Micros ESV 60 hematology cell counter (Horiba Medical, USA).

### 2.4 Statistical analysis

Two-way analysis of variance (two-way ANOVA) was done. P values for the main effects (type of diet [CD or HFD] and genotype [wild-type or *Hamp1*^*−/−*^] or duration of feeding) and interactions were reported. Where a significant interaction between the 2 independent variables (diet and genotype) was observed, post-hoc pair-wise comparisons were made using Mann-Whitney *U* test. Statistical Package for Social Scientists (SPSS), version 21.0, was used for all statistical analyses.

## 3 Results

### 3.1 Effect of HFD-feeding, for varying periods, on erythroid differentiation in wild-type mice

**Fig. 1:**
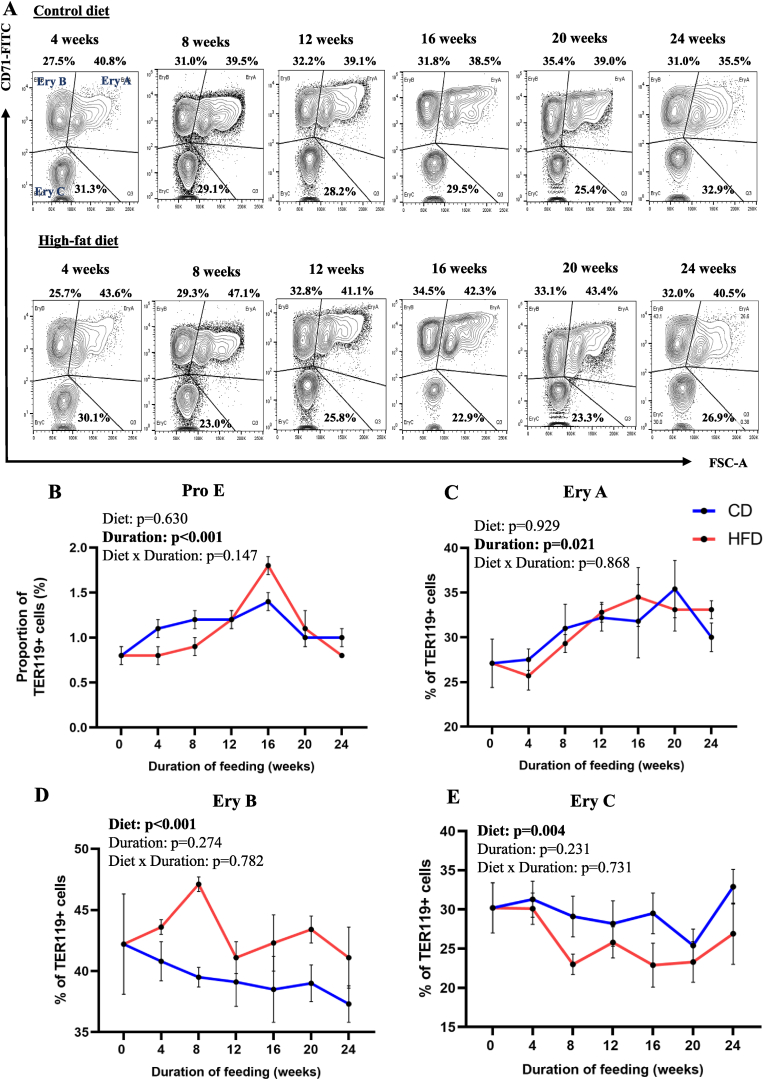
Effect of HFD-feeding, for varying periods, on TED in the bone marrow from WT mice (based on CD71 expression) (A) Ter119-positive cells in the bone marrow (from WT mice fed CD or HFD for varying periods) were resolved into 4 distinct populations (pro-erythroblasts, EryA [early erythroid precursors], EryB [late erythroid precursors] and EryC [mature erythrocytes]), based on CD71 expression and cell size. (B–E) Quantification of changes in proportions of Pro-E, EryA, EryB and EryC populations (expressed as percentage of Ter119-positive cells) in WT mice fed CD and HFD for varying periods (as indicated). Data are shown as means ± SE; n = 3–6 at all time points. Two-way ANOVA was used to analyze the data.

Results of flow cytometric analyses of Ter119-positive (erythroid) cells in the bone marrow of WT mice fed CD or HFD (for 4, 8, 12, 16, 20, and 24 weeks), using CD71 expression and cell size (FSC) as maturation markers, are shown in Fig. 1A. The duration of feeding significantly affected proportions of ProE (pro-erythroblasts) (Fig. 1B) and early erythroid precursors (EryA) (polychromatic and basophilic erythroblasts) (Fig. 1C), but not the type of diet. On the other hand, the proportion of late erythroid precursors (EryB) (consisting of orthochromatic erythroblasts and reticulocytes) (Fig. 1D) and mature RBCs (EryC) (Fig. 1E) in the bone marrow these mice were significantly affected by the type of diet, but not its duration.

**Fig. 2:**
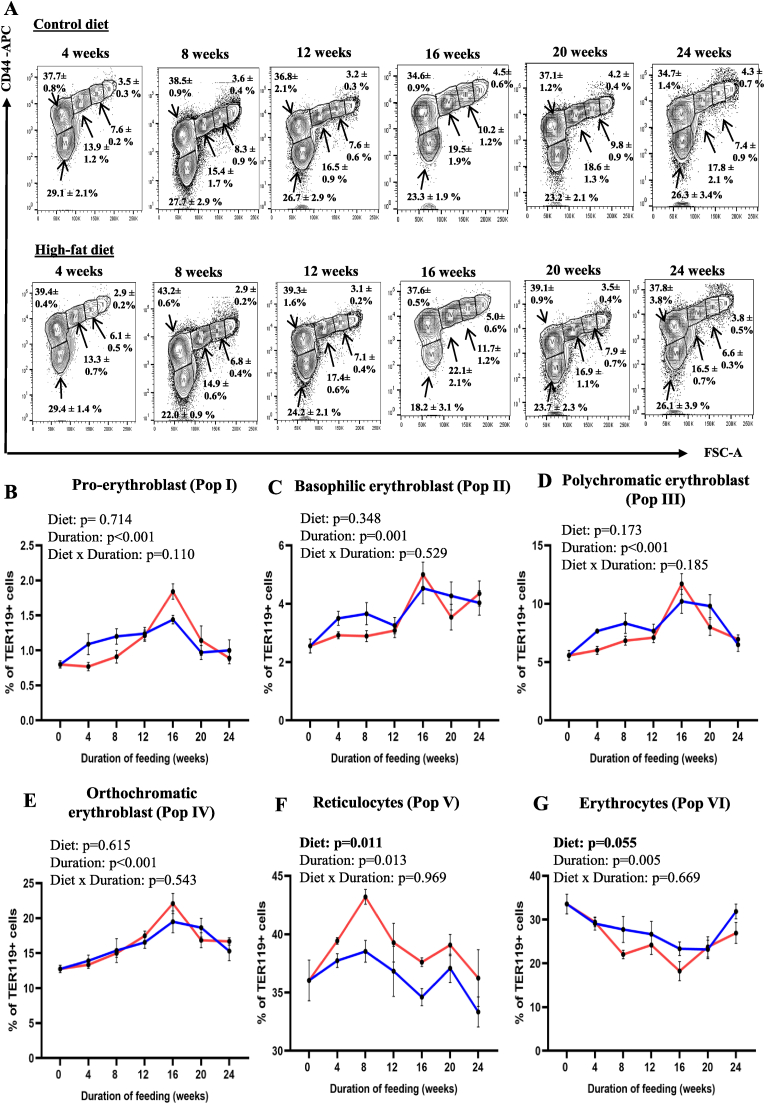
Effect of HFD-feeding, for varying periods, on TED in the bone marrow from WT mice (based on CD44 expression) (A) TER119-positive cells in the bone marrow (from WT mice fed CD or HFD for varying periods) were resolved into 6 distinct populations of erythroblasts (pro-, basophilic, polychromatic and orthochromatic erythroblasts, reticulocytes and mature erythrocytes), based on CD44 expression and cell size. (B–G) Proportions of pro-, basophilic, polychromatic and orthochromatic erythroblasts, reticulocytes and mature erythrocytes (expressed as percentage of Ter119-positive cells) in the bone marrow from WT mice fed CD and HFD for varying periods (as indicated). Data are shown as means ± SE; n = 3–6. Two-way ANOVA was used to analyze the data.

Further analyses of erythroid maturation, using CD44 expression as the maturation marker (Fig. 2A), showed that the duration of feeding had significant effects on the proportions of early precursors (Pop I, II and III representing pro-erythroblasts, basophilic and polychromatic erythroblasts, respectively) (Fig. 2B–D) and late precursors (Pop IV [orthochromatic erythroblasts]) (Fig. 2E) and on proportions of reticulocytes and erythrocytes (Fig. 2F and G). The proportion of reticulocytes in the bone marrow were also significantly affected by the type of diet, with it being higher in the HFD-fed group (Fig. 2F).

### 3.2 Effect of HFD on erythroid differentiation in *Hamp1*^*−/−*^ mice

At baseline (in mice aged 8 weeks and prior to starting CD/HFD feeding), wild-type (WT) and *Hamp1*^*−/−*^ mice had similar hemoglobin concentrations (Suppl Fig. 5A). The total number of erythroid cells (Ter119+ cells) in the bone marrow (Suppl Fig. 5B) and the proportions of the various erythroid precursors (Suppl Figs. 5C and D) were also similar in the 2 groups of mice.

**Fig. 3:**
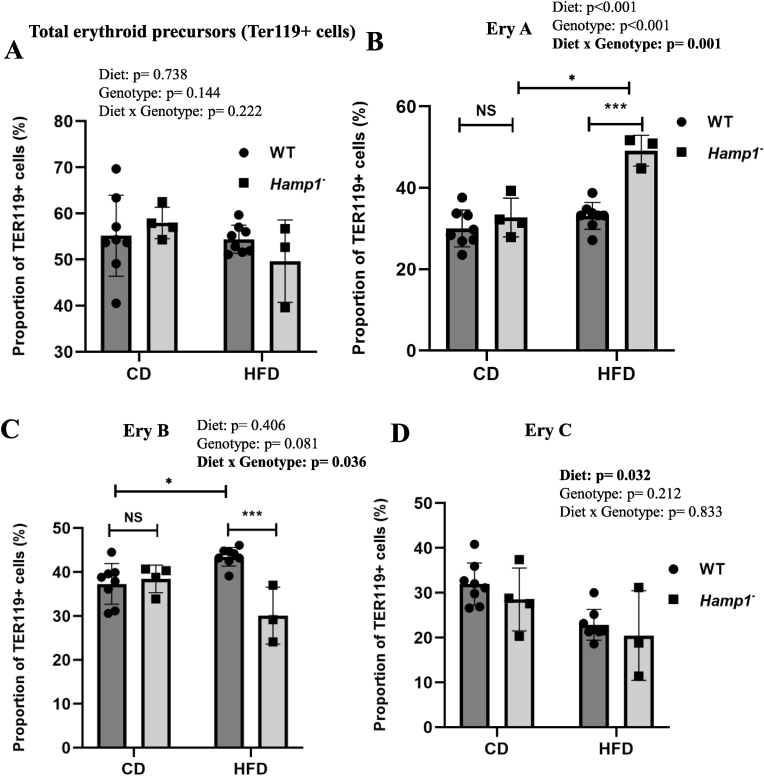
Effect of HFD-feeding on erythroid maturation in wild-type and Hamp1^−/−^ mice (populations resolved based on CD71 expression). Wild-type (WT) and *Hamp1*^*−/−*^ mice were fed CD or HFD for 24 weeks. The proportion of Ter119-positive cells (erythroid precursors) in the bone marrow (**A**), and its subpopulations, viz., EryA (early erythroid precursors) (**B**), EryB (late erythroid precursors) (**C**), and EryC (mature erythrocytes) (**D**) are shown. Data are shown as means ± SE; n = 8 each for WT mice fed CD and HFD, n = 4 for *Hamp1*^*−/−*^ mice fed CD and n = 3 for *Hamp1*^*−/−*^ mice fed HFD. Statistical analysis was carried out using two-way ANOVA.Where statistically significant interactions were found, the Mann Whitney *U* test was used for post-hoc pairwise comparisons.

**Fig. 4:**
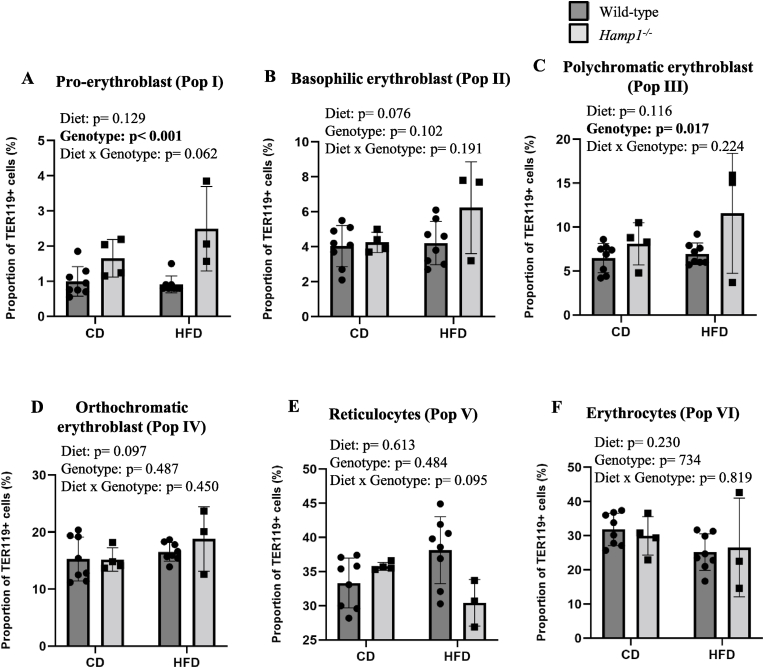
**Effect of HFD-feeding on erythroid maturation in wild-type and Hamp1−/− mice (populations resolved based on CD44 expression)**. Wild-type (WT) and Hamp1^−/−^ mice were fed CD or HFD for 24 weeks. Bone marrow TER119-positive cells (erythroid precursors) were resolved into their subpopulations based on CD44 expression, viz., pro-erythroblast (**A**), basophilic (**B**), polychromatic (**C**) and orthochromatic (**D**) erythroblasts, reticulocytes (**E**) and mature erythrocytes (**F**), as shown. Data are shown as means ± SE; n = 8 for WT mice fed CD or HFD, n = 4 for Hamp1^−/−^ mice fed CD and n = 3 for *Hamp1*^*−/−*^ mice fed HFD. Statistical analysis was done by two-way ANOVA.

Wild-type and *Hamp1*^*−/−*^ mice fed CD and HFD for 24 weeks had similar proportions of Ter119-positive cells in the bone marrow (Fig. 3A). However, the EryA population was significantly higher in the HFD-fed *Hamp1*^*−/−*^ mice than in HFD-fed WT mice and in CD-fed *Hamp1*^*−/−*^ mice (Fig. 3B). The EryB population was significantly higher in HFD-fed WT mice than in those fed CD (as described earlier) and in HFD-fed *Hamp1*^*−/−*^ mice (Fig. 3C). HFD-feeding had a significant effect on the EryC population, irrespective of genotype (Fig. 3D). CD44-based resolution of erythroid precursors showed significant effects of genotype only on pro- (Fig. 4A) and polychromatic (Fig. 4C) erythroblasts. The type of diet did not significantly affect any of the cell populations (Fig. 4). No significant interactions were noted between the diet fed and genotype.

### 3.3 Hematological parameters

As shown in Table 1, at the end of 24 weeks of feeding, *Hamp1*^*−/−*^ mice had significantly higher values for hemoglobin, RBC count, hematocrit, MCH and MCV than wild-type mice. These were not affected by the type of diet fed. Hemoglobin levels in the WT mice fed HFD were lower than in those fed CD (13.48 ± 0.29 g/dL vs. 14.13 ± 0.70 g/dL) (Table 1), after 24 weeks of feeding, but not significantly so (p = 0.062, student t-test). The reticulocyte count in peripheral blood could not be determined to confirm whether there was a decrease in the reticulocytes in blood, as it was not possible to carry out this estimation in the hematology analyser that was used for estimations done on the mouse blood samples.

**Table 1:** Peripheral blood counts of wild-type and Hamp1^−/−^ mice fed CD or HFD for 24 weeks.

	*Wild type*		*Hamp1*^*−/−*^		*P value* *2-way ANOVA*		
	CD (n = 6)	HFD (n = 6)	CD (n = 8)	HFD (n = 6)	Main Effects		Genotype∗ Diet
					Genotype	Diet	
Hemoglobin (g/dl)	14.13 ± 0.70	13.48 ± 0.29	15.70 ± 0.73	16.50 ± 1.58	**<0.001**	0.841	0.062
WBC count (10^3^/mm^3^)	5.23 ± 1.95	5.98 ± 0.9	5.53 ± 1.66	6.78 ± 1.65	0.396	0.125	0.691
RBC count (10^3^/mm^3^)	9.35 ± 0.61	9.10 ± 0.80	9.92 ± 0.78	9.96 ± 1.21	**0.049**	0.752	0.660
Platelet count (10^3^/mm^3^)	767.5 ± 99.7	617.2.6 ± 237.5	640.9 ± 154.9	754.0 ± 146.4	0.939	0.780	0.057
HCT (%)	37.67 ± 3.57	36.18 ± 4.55	43.41 ± 5.80	44.40 ± 7.72	**0.005**	0.913	0.585
MCV (fL)	40.17 ± 1.83	39.83 ± 2.14	43.63 ± 3.02	44.33 ± 3.67	**0.002**	0.866	0.641
MCH (pg)	15.13 ± 1.30	14.92 ± 1.27	15.90 ± 1.05	16.62 ± 0.58	**0.009**	0.566	0.289
MCHC (%)	37.83 ± 4.92	37.73 ± 4.75	36.66 ± 4.29	37.71 ± 4.45	0.746	0.795	0.753

Blood counts were done in peripheral blood collected from wild-type and Hamp1−/− mice fed control diet (CD) or high-fat diet (HFD) for a period of 24 weeks. Data were presented as mean ± SD.

## 4 Discussion

In our earlier publication, we have described the effects of high-fat feeding on systemic iron homeostasis, using the same mouse model as in the present study [21]. Our results showed that the onset of insulin resistance (at 12 weeks) preceded the onset of changes in systemic iron homeostasis. For example, hepatic iron stores progressively declined from 16 weeks onwards and this was associated with an increase in total adipose tissue iron content. Serum iron levels and markers of systemic inflammation were not significantly different between HFD- and CD-fed mice. Gene expression of duodenal proteins involved in iron absorption, divalent metal transporter-1, ferroportin and duodenal cytochrome *b*, was also not affected by HFD-feeding at any of the timepoints studied. We also showed that serum levels of erythropoietin were not significantly affected by HFD-feeding. Gene expression of erythroferrone, the erythroid regulator of hepcidin [29], in the bone marrow, was also not affected by HFD-feeding at any of the time points studied. Overall, these observations are in keeping with the findings reported in the present paper that HFD-feeding did not significantly affect TED in the bone marrow of these mice. Serum hepcidin levels were significantly lower in HFD-fed mice than in CD-fed mice, after 20 and 24 weeks of feeding, but not at earlier time points. However, the absence of up-regulation of erythroferrone suggests that the lowered hepcidin does not appear to be mediated by enhanced erythropoiesis in the bone marrow. This observation too is in keeping with the results reported in the present paper.

It has been reported that the presence of IR in human subjects, especially in the context of the metabolic syndrome, was associated with increases in hemoglobin levels, RBC count and hematocrit [[5], [6], [7], [8], [9], [10], [11]]. Since insulin has been shown to have a tropic effect on erythroid maturation and differentiation *in vitro* [[12], [13], [14], [15]], the stimulatory effect of IR-induced hyperinsulinemia was suggested to mediate this effect *in vivo* [5]. On the other hand, obesity has been reported to be associated with iron deficiency. This is thought to be due to the effect of low-grade systemic inflammation, seen in obesity, in inducing hepcidin with resultant decreases in intestinal iron absorption [[30], [31], [32], [33]].

In the present study, wild-type mice fed HFD for up to 24 weeks developed IR and hyperinsulinemia (as we have shown earlier [21]) but, overall, did not show evidence of enhanced erythropoiesis (Fig. 1, Fig. 2), or alterations in hematological parameters in peripheral blood (Table 1). This suggests that IR or hyperinsulinemia *per se* did not produce significant effects on erythropoiesis in this model. This indicates that there may be factors unrelated to IR that may underlie the hematological effects reported in human subjects. Examples of such factors may include the effects of inflammatory cytokines and adipokines released by adipose tissue and by cells of the innate immune system, resulting in chronic low-grade systemic inflammation [34,35], which is known to affect erythropoiesis and iron metabolism [36]. In addition, the effects of adipokines derived from bone marrow adipocytes on hematopoiesis is an area that has not been extensively investigated [37,38]. Given that there are limitations to the use of animal models to replicate human biology, studies on patients with insulin resistance (type 2 diabetes mellitus/metabolic syndrome) would be necessary to further elucidate the possible role of the above-mentioned mechanisms.

Although HFD-feeding did not alter the proportion of early and late erythroid precursors in wild-type mice, there was a significantly higher number of reticulocytes in the bone marrow (Fig. 1, Fig. 2F). Reticulocytes are formed following enucleation of orthochromatic erythroblasts. The deformability of the reticulocyte membrane plays an important role in their release into circulation [39]. Early reticulocytes in the bone marrow have relatively high membrane stiffness, which decreases progressively over a period of 24h, after which they are sufficiently flexible to negotiate the narrow apertures of the bone marrow sinusoids [40]. Therefore, a decrease in membrane deformability, due to any condition that affects this complex process, can result in the retention of reticulocytes within the marrow. Chronic hyperinsulinemia has been reported to be associated with decreased membrane fluidity in erythrocytes [[41], [42], [43], [44]]. It is possible that such changes may also affect developing erythroblasts, resulting in the formation of reticulocytes with increased membrane stiffness. This may result in impaired release of reticulocytes into circulation, thus increasing the proportion of reticulocytes in the marrow, as seen in this study. Impaired release would be expected to lead to a decrease in the number of reticulocytes in peripheral blood. However, we could not estimate this (as mentioned in the methodology section) and, hence, are unable to substantiate this postulate. However, hemoglobin levels in the WT mice fed HFD were lower than in those fed CD (13.48 ± 0.29 g/dL vs. 14.13 ± 0.70 g/dL) (Table 1), after 24 weeks of feeding, but not significantly so (p = 0.062, student t-test). It is possible that impaired reticulocyte maturation and/or their release from the bone marrow may explain the lower hemoglobin level in peripheral blood in the HFD-fed WT mice. However, further work would be required to confirm/refute this possibility.

At the end of 24 weeks of feeding, *Hamp1*^*−/−*^ mice showed significantly higher values for hemoglobin, RBC counts, hematocrit, MCV, and MCH than corresponding values in wild-type mice. This was seen irrespective of the type of diet fed, suggesting enhanced erythropoiesis in the *Hamp1*^*−/−*^ mice (Table 1). Interestingly, the increase in early erythroid precursors in the bone marrow was more prominent in HFD-fed *Hamp1*^*−/−*^ mice and not in those fed CD (Fig. 3, Fig. 4A). We have shown in our earlier work that, unlike wild-type mice, *Hamp1*^*−/−*^ mice fed an HFD did not develop hyperinsulinemia [22]. In fact, insulin levels in these mice were found to be significantly lower than in WT mice fed the HFD and were similar to levels in *Hamp1*^*−/−*^ mice on the CD. The mechanism that may underlie this effect is currently unclear. Further work would be required to clarify this.

In summary, we show that HFD-feeding in WT mice did not significantly affect early erythroid maturation but resulted in higher number of reticulocytes in the bone marrow. *Hamp1*^*−/−*^ mice (aged 32 weeks) had higher hemoglobin, hematocrit, MCV, and MCH values than wild-type mice of the same age, irrespective of the diet fed. *Hamp1*^*−/−*^ mice fed HFD for 24 weeks had higher numbers of early erythroid precursors in the bone marrow.

## CRediT authorship contribution statement

**Joe Varghese:** Writing – review & editing, Writing – original draft, Visualization, Methodology, Investigation, Funding acquisition, Formal analysis, Conceptualization. **Jithu James Varghese:** Writing – review & editing, Writing – original draft, Visualization, Methodology, Investigation, Formal analysis, Data curation. **Molly Jacob:** Writing – review & editing, Validation, Supervision, Project administration, Funding acquisition, Formal analysis, Conceptualization.

## Ethics approval

Institutional Animal Ethics Committee at Christian Medical College, Vellore, India (IAEC No. 14/2013 and 8/2014).

## Declaration of generative AI and AI-assisted technologies in the writing process

Generative AI and AI-assisted technologies were not used in the writing process.

## Funding

This work was supported by the 10.13039/501100009053Wellcome Trust/DBT India Alliance Fellowship/Grant (Ref no. 500190/Z/11/Z) awarded to JV and a grant awarded by the 10.13039/501100001843Science and Engineering Research Board (10.13039/501100001843SERB), Government of India (EMR/2015/0 0 0502) to MJ.

## Declaration of competing interest

The authors declare the following financial interests/personal relationships which may be considered as potential competing interests:

Molly Jacob reports financial support was provided by 10.13039/501100001843Science and Engineering Research Board. Joe Varghese reports financial support was provided by 10.13039/501100009053Wellcome Trust DBT India Alliance. If there are other authors, they declare that they have no known competing financial interests or personal relationships that could have appeared to influence the work reported in this paper.

## Data Availability

Data will be made available on request.
