# Supplementary material for: Effect of a high-fat diet and iron overload on erythropoiesis in mice
Source: Biochem Biophys Rep. 2025 Feb 1;41:101919. doi: 10.1016/j.bbrep.2025.101919 (PMC11841077; doi:10.1016/j.bbrep.2025.101919)
Supplement: Multimedia component 1 [file mmc1.docx]

*Supplementary Methods*

*1. Flow cytometric analyses*

Flow cytometric analysis was carried out in accordance with published protocols (1,2), to phenotype the bone marrow cells isolated, to delineate the stages of TED.

*1..1. Staining of cells*

For staining, 1 x 10^6^ cells of each sample were re-suspended in 200 µL of ice-cold staining buffer, containing rat anti-mouse CD16/CD32 blocking antibody (cat. no. 553142, BD Biosciences, USA) at a concentration of 25 µg/mL. An adequate volume of an antibody pre-mix, consisting of fluorochrome-conjugated antibodies, was prepared, as shown in Table 1 below.

Two hundred microliters of the antibody pre-mix (containing all three antibodies) were added to each of the tubes containing the samples of cells, and mixed gently by inverting the tubes.

**Table 1: Antibodies used for flow cytometric analyses**

| **Antibody (fluorochrome)** | **Clone** | **Reactivity** | **Supplier (cat.no.)** | **Conc. (mg/mL)** | **Dilution used** |
| --- | --- | --- | --- | --- | --- |
| CD71 (FITC) | C2 | Mouse | BD Pharmingen, USA (#553266) | 0.5 mg/ml | 1:200 |
| TER-119 (PE) | TER-119 | Mouse | BD Pharmingen, USA (#553673) | 0.2 mg/ml | 1:200 |
| C44 (APC) | IM7 | Mouse | BD Pharmingen, USA (#559250) | 0.2 mg/ml | 1:200 |

FITC – Fluorescein isothiocyanate; PE – Phycoerythrin; APC – Allophycocyanin

*1.2. Preparation of control samples*

A mixture of cells was prepared, taking aliquots from all the samples available for analysis on a particular day, and used for preparation of control samples as follows:Unstained controls: 1 x 10^6^ cells were re-suspended in 400 µL of staining buffer.

- ‘Single-stained’ controls: 1 x 10^6^ cells were re-suspended in 200 µL of staining buffer. Each antibody (diluted as shown in Table 1) (200 µL) was added to one such suspension. Thus, there was one ‘single-stained’ control for each of the 3 antibodies used.
- ‘Fluorescence minus one' or FMO controls: 1 x 10^6^ cells were re-suspended in 200 µL of staining buffer and 200 µL of an antibody pre-mix containing all the antibodies, except one, was added. Thus, FMO for CD71-FITC consisted of an antibody pre-mix containing anti-TER119 (PE) and anti-CD44 (APC), but not CD71 (FITC). Similarly, FMO for anti-TER119 (PE) contained anti-CD44 (APC) and anti-CD71 (FITC), but not anti-TER119 (PE) and FMO for anti-CD44 (APC) contained anti-CD71 (FITC) and anti-TER119 (PE), but not anti-CD44 (APC).

*1.3. Incubations*

All the sample and control tubes were incubated for 1 hour in the dark at 4°C. At the end of the incubations, 3 mL of staining buffer was added to each tube; the contents were subjected to centrifugation at 400*g* for 5 min at 4°C. The washing step was repeated; cells were finally re-suspended in 500 μL of staining buffer and used for flow cytometric analyses.

*1.4 Data acquisition*

The samples were analyzed using a BD FACS Aria III instrument with 5 lasers (561-nm [Y/G], 488-nm [Blue], 633-nm [Red], 405-nm [Violet], and 375-nm [UV]) and 11 colors, at the Core Facility, Center for Stem Cell Research, CMC, Vellore, India. BD FACS Diva software was used for operating the instrument.

**References**

1. Liu J, Zhang J, Ginzburg Y, Li H, Xue F, De Franceschi L, et al. Quantitative analysis of murine terminal erythroid differentiation in vivo: novel method to study normal and disordered erythropoiesis. Blood. 2013 Feb 21;121(8):e43-49.

2. Liu Y, Pop R, Sadegh C, Brugnara C, Haase VH, Socolovsky M. Suppression of Fas-FasL coexpression by erythropoietin mediates erythroblast expansion during the erythropoietic stress response in vivo. Blood. 2006 Jul 1;108(1):123–33.
