# Supplementary material for: Effect of a high-fat diet and iron overload on erythropoiesis in mice
Source: Biochem Biophys Rep. 2025 Feb 1;41:101919. doi: 10.1016/j.bbrep.2025.101919 (PMC11841077; doi:10.1016/j.bbrep.2025.101919)
Supplement: Multimedia component 2 [file mmc2.pptx]

## Slide 1
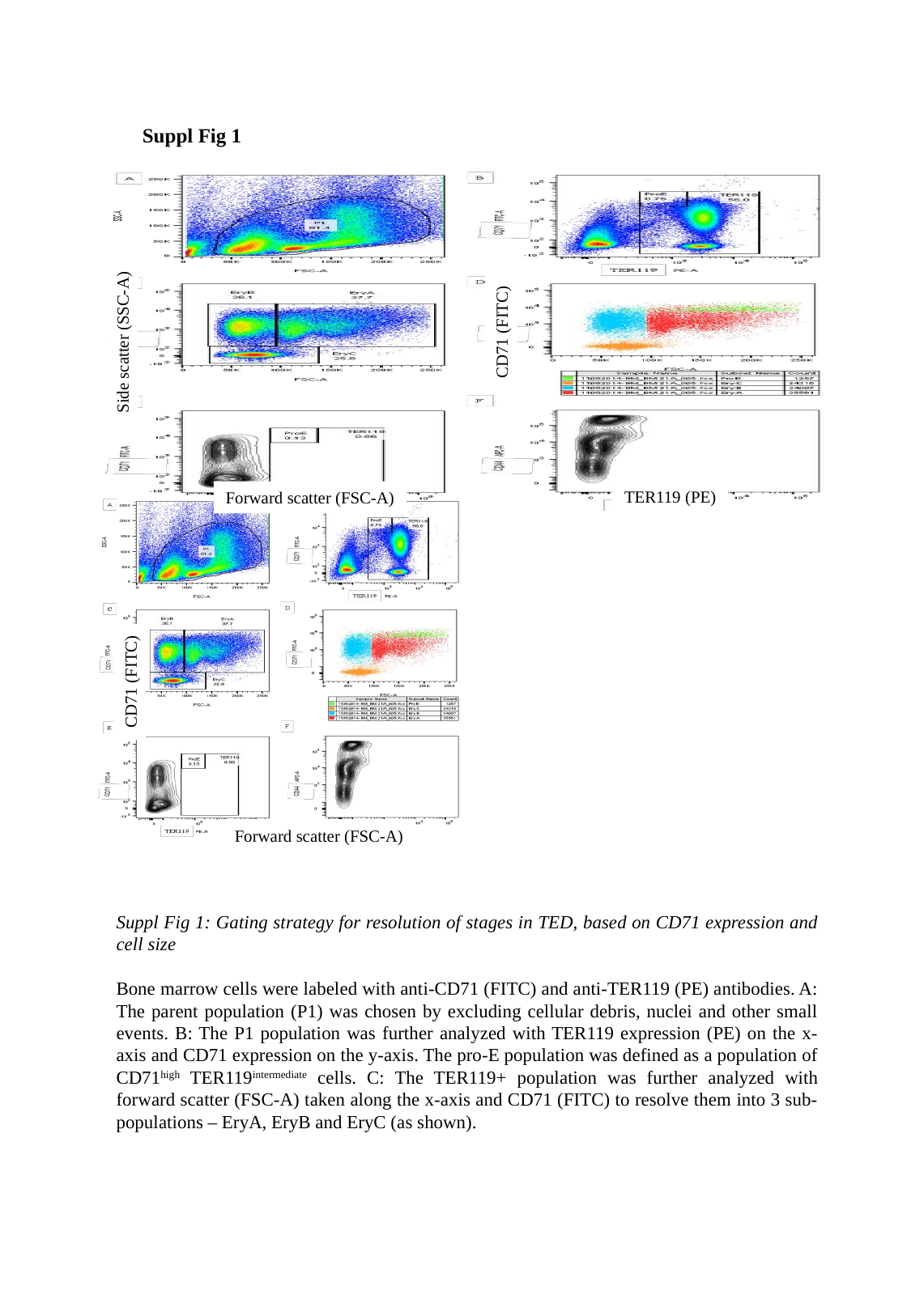

Suppl Fig 1
CD71 (FITC)
Side scatter (SSC-A)
TER119 (PE)
Forward scatter (FSC-A)
CD71 (FITC)
Forward scatter (FSC-A)
Suppl Fig 1: Gating strategy for resolution of stages in TED, based on CD71 expression and cell size
Bone marrow cells were labeled with anti-CD71 (FITC) and anti-TER119 (PE) antibodies. A: The parent population (P1) was chosen by excluding cellular debris, nuclei and other small events. B: The P1 population was further analyzed with TER119 expression (PE) on the x-axis and CD71 expression on the y-axis. The pro-E population was defined as a population of CD71high TER119intermediate cells. C: The TER119+ population was further analyzed with forward scatter (FSC-A) taken along the x-axis and CD71 (FITC) to resolve them into 3 sub-populations – EryA, EryB and EryC (as shown).
