# Supplementary material for: Effect of a high-fat diet and iron overload on erythropoiesis in mice
Source: Biochem Biophys Rep. 2025 Feb 1;41:101919. doi: 10.1016/j.bbrep.2025.101919 (PMC11841077; doi:10.1016/j.bbrep.2025.101919)
Supplement: Multimedia component 3 [file mmc3.pptx]

## Slide 1
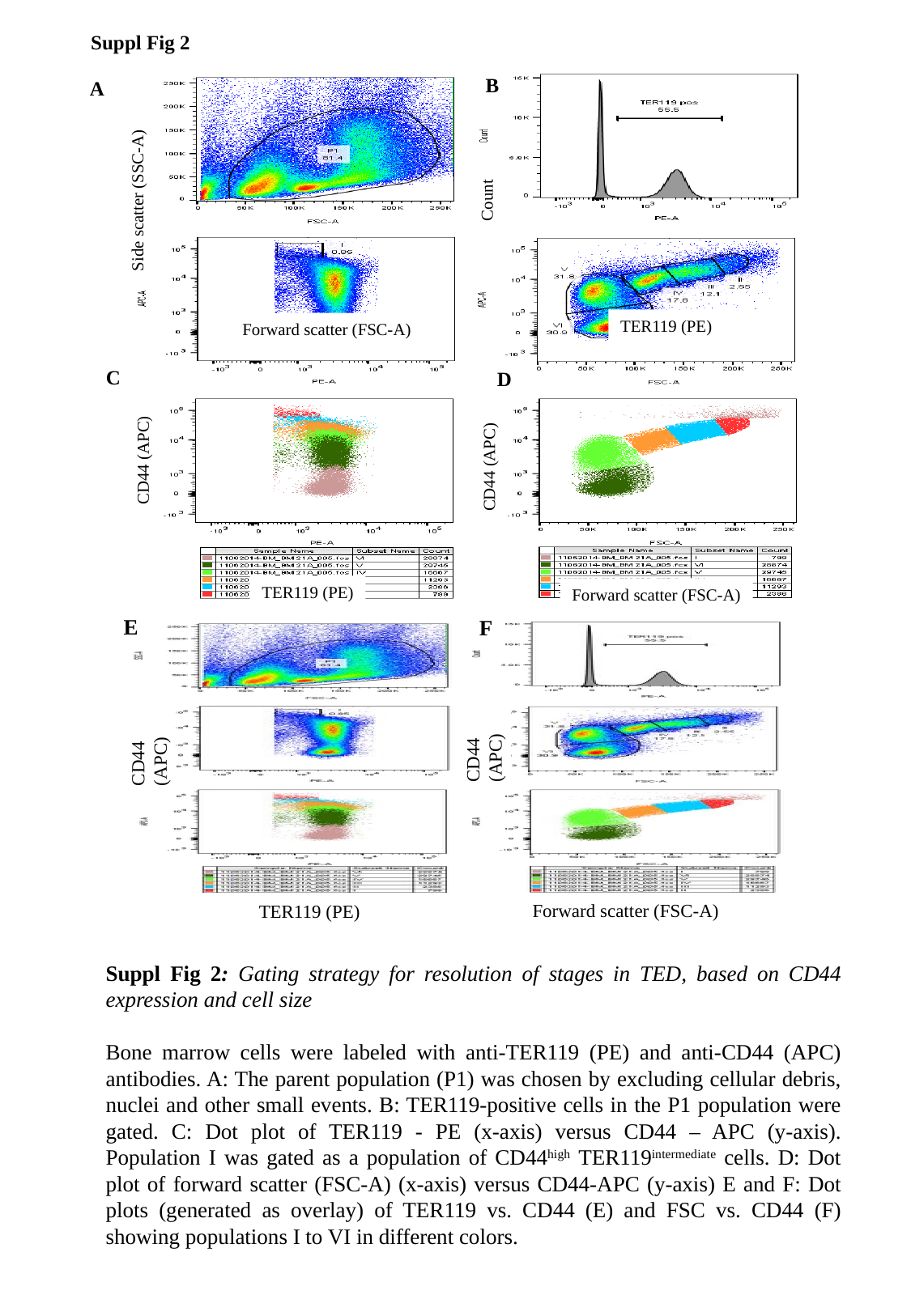

Suppl Fig 2
B
A
Count
Side scatter (SSC-A)
TER119 (PE)
Forward scatter (FSC-A)
C
D
CD44 (APC)
CD44 (APC)
TER119 (PE)
Forward scatter (FSC-A)
E
F
CD44 (APC)
CD44 (APC)
Forward scatter (FSC-A)
TER119 (PE)
Suppl Fig 2: Gating strategy for resolution of stages in TED, based on CD44 expression and cell size
Bone marrow cells were labeled with anti-TER119 (PE) and anti-CD44 (APC) antibodies. A: The parent population (P1) was chosen by excluding cellular debris, nuclei and other small events. B: TER119-positive cells in the P1 population were gated. C: Dot plot of TER119 - PE (x-axis) versus CD44 – APC (y-axis). Population I was gated as a population of CD44high TER119intermediate cells. D: Dot plot of forward scatter (FSC-A) (x-axis) versus CD44-APC (y-axis) E and F: Dot plots (generated as overlay) of TER119 vs. CD44 (E) and FSC vs. CD44 (F) showing populations I to VI in different colors.
