# Supplementary material for: Effect of a high-fat diet and iron overload on erythropoiesis in mice
Source: Biochem Biophys Rep. 2025 Feb 1;41:101919. doi: 10.1016/j.bbrep.2025.101919 (PMC11841077; doi:10.1016/j.bbrep.2025.101919)
Supplement: Multimedia component 4 [file mmc4.pptx]

## Slide 1
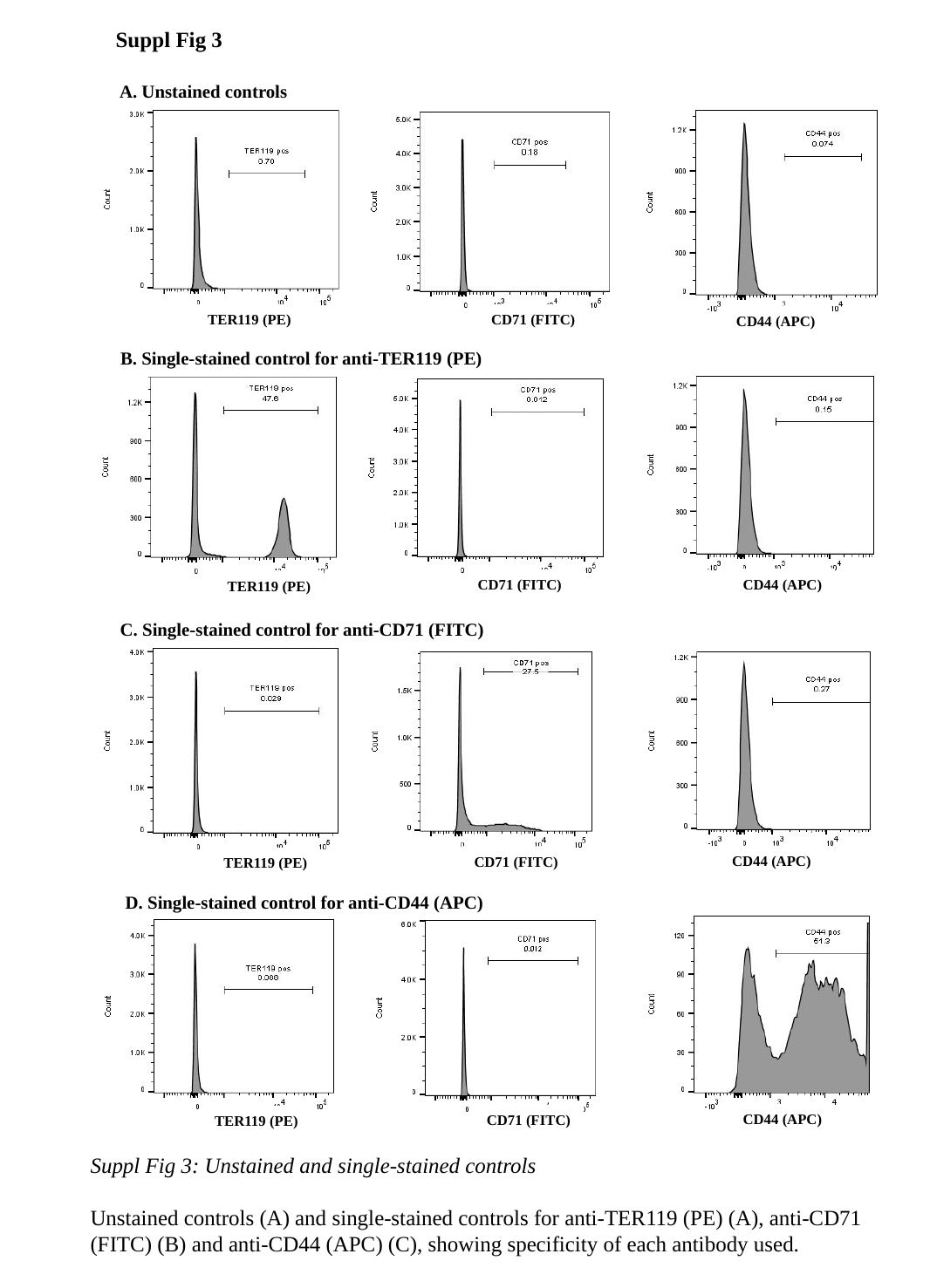

Suppl Fig 3
A. Unstained controls
TER119 (PE)
CD71 (FITC)
CD44 (APC)
B. Single-stained control for anti-TER119 (PE)
CD44 (APC)
CD71 (FITC)
TER119 (PE)
C. Single-stained control for anti-CD71 (FITC)
CD44 (APC)
CD71 (FITC)
TER119 (PE)
D. Single-stained control for anti-CD44 (APC)
CD44 (APC)
CD71 (FITC)
TER119 (PE)
Suppl Fig 3: Unstained and single-stained controls
Unstained controls (A) and single-stained controls for anti-TER119 (PE) (A), anti-CD71 (FITC) (B) and anti-CD44 (APC) (C), showing specificity of each antibody used.
