# Supplementary material for: Effect of a high-fat diet and iron overload on erythropoiesis in mice
Source: Biochem Biophys Rep. 2025 Feb 1;41:101919. doi: 10.1016/j.bbrep.2025.101919 (PMC11841077; doi:10.1016/j.bbrep.2025.101919)
Supplement: Multimedia component 5 [file mmc5.pptx]

## Slide 1
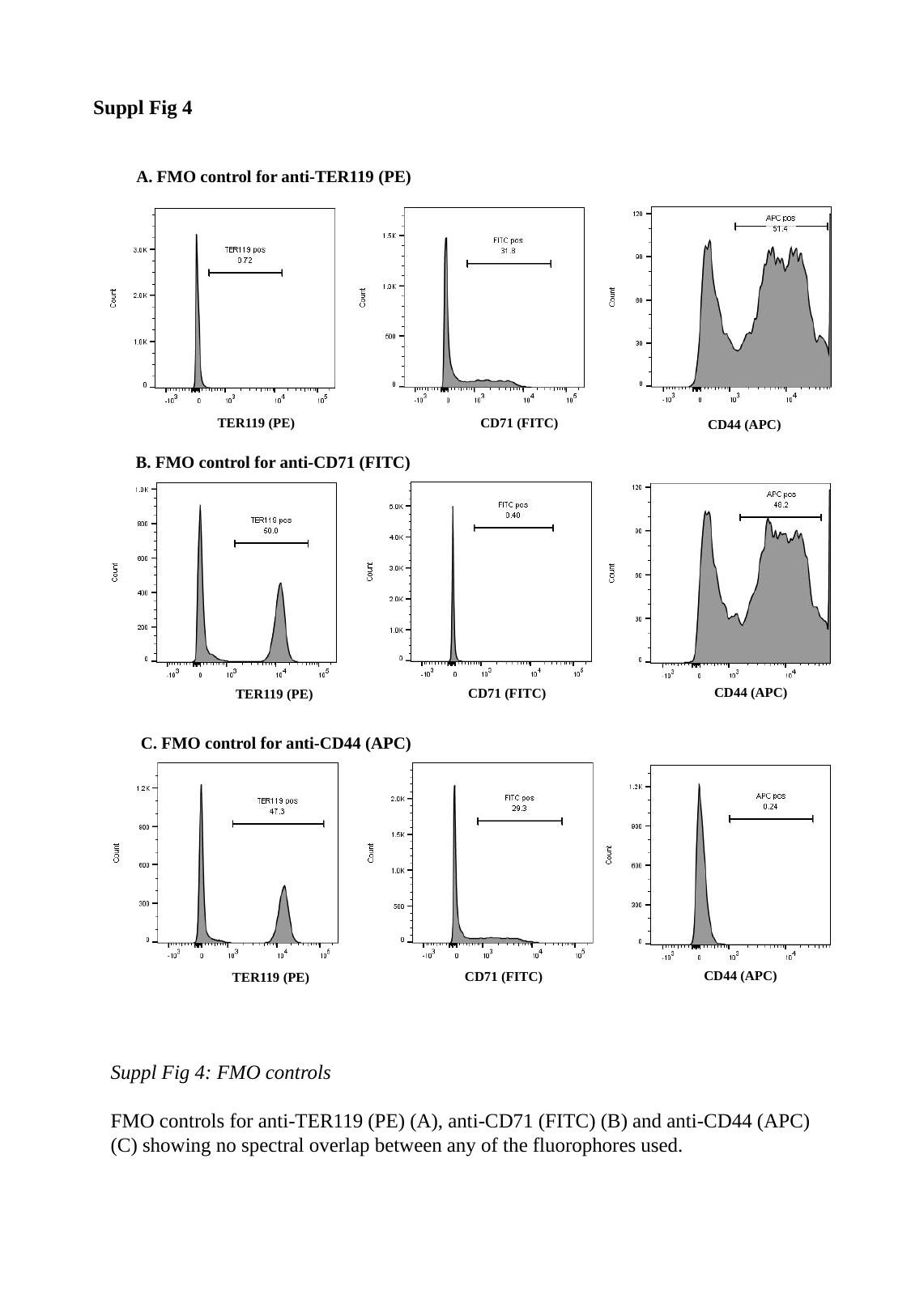

Suppl Fig 4
A. FMO control for anti-TER119 (PE)
TER119 (PE)
CD71 (FITC)
CD44 (APC)
B. FMO control for anti-CD71 (FITC)
CD44 (APC)
CD71 (FITC)
TER119 (PE)
C. FMO control for anti-CD44 (APC)
CD44 (APC)
CD71 (FITC)
TER119 (PE)
Suppl Fig 4: FMO controls
FMO controls for anti-TER119 (PE) (A), anti-CD71 (FITC) (B) and anti-CD44 (APC) (C) showing no spectral overlap between any of the fluorophores used.
