# Supplementary material for: Effect of a high-fat diet and iron overload on erythropoiesis in mice
Source: Biochem Biophys Rep. 2025 Feb 1;41:101919. doi: 10.1016/j.bbrep.2025.101919 (PMC11841077; doi:10.1016/j.bbrep.2025.101919)
Supplement: Multimedia component 6 [file mmc6.pptx]

## Slide 1
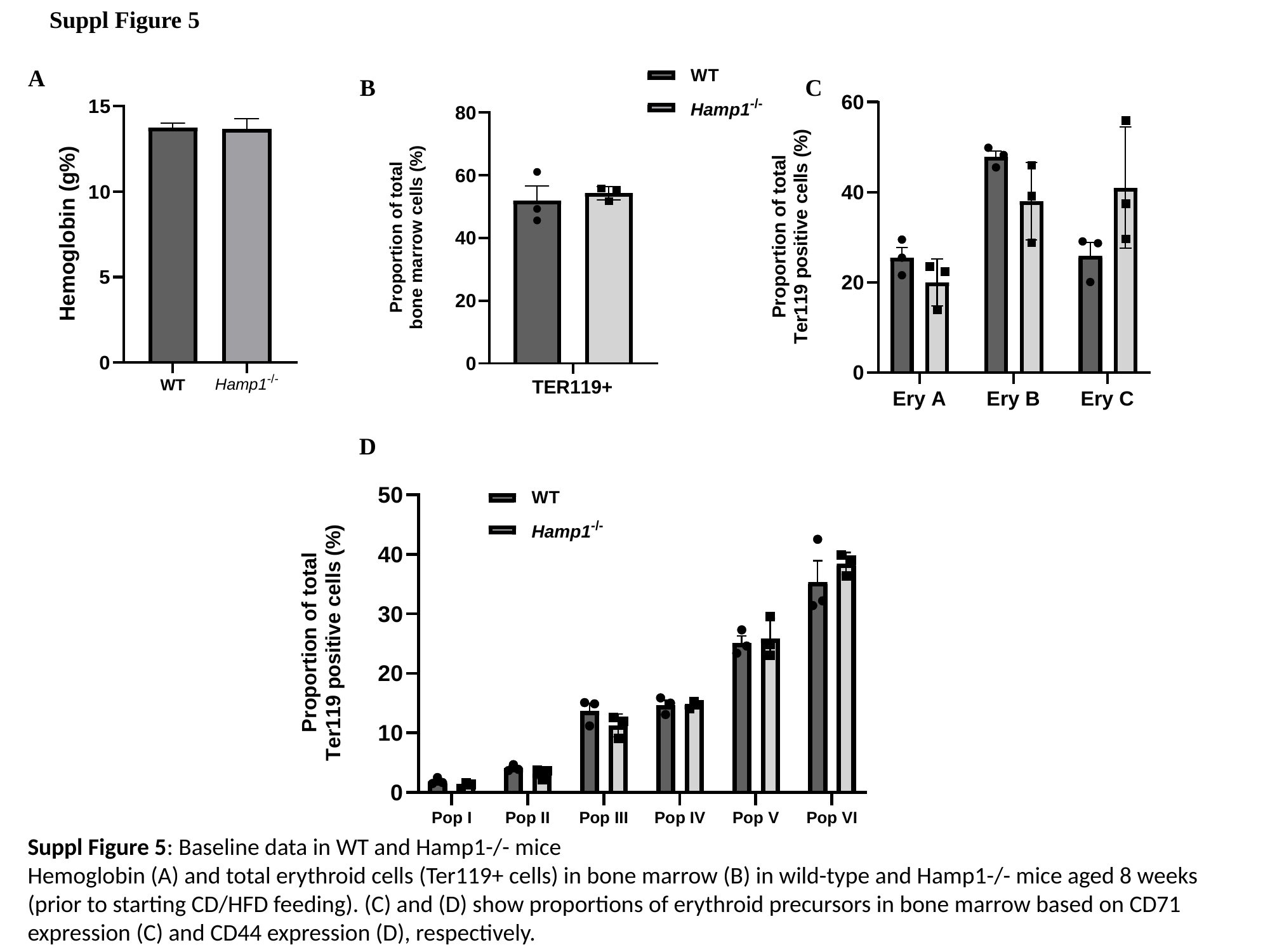

Suppl Figure 5
A
B
C
D
Suppl Figure 5: Baseline data in WT and Hamp1-/- mice
Hemoglobin (A) and total erythroid cells (Ter119+ cells) in bone marrow (B) in wild-type and Hamp1-/- mice aged 8 weeks (prior to starting CD/HFD feeding). (C) and (D) show proportions of erythroid precursors in bone marrow based on CD71 expression (C) and CD44 expression (D), respectively.
